# Circadian and Geotactic Behaviors: Genetic Pleiotropy in *Drosophila Melanogaster*

**DOI:** 10.5334/jcr.140

**Published:** 2016-06-24

**Authors:** Dale L. Clayton

**Affiliations:** 1 Department of Biology, La Sierra University, 4500 Riverwalk Parkway, Riverside, CA

**Keywords:** gene-pleiotropy, Drosophila, geotaxis, circadian, *cry*, *Pdf*, *tau*

## Abstract

Data presented in this paper test the hypotheses that Hirsch’s positive geotaxis *(Lo)* and negative geotaxis *(Hi5)* strains of Drosophila melanogaster (fruit fly) differ in length of the free-running circadian activity period (tau) as well as adult geotaxis.

Several genes have been shown to alter geotaxis in *Drosophila*. Two of these genes, cryptochrome *(cry)* and Pigment-dispersing-factor *(Pdf)* are integral to the function of biological clocks. Pdf plays a crucial role in maintaining free-running circadian periods. The *cry* gene alters blue-light (<420 nm) phototransduction which affects biological clocks, spatial orientation and taxis relative to gravity, magnetic fields, solar, lunar, and celestial radiation in several species. The cry gene is involved in phase resetting (entrainment) of the circadian clock by blue light (<420 nm).

Geotaxis involves spatial orientation, so it might be expected that geotaxis is linked genetically with other forms of spatial orientation. The association between geotaxis and biological clocks is less intuitive. The data and the literature presented here show that genes, physiology and behavioural aspects of geotaxis, biological clocks, magnetosensitivity and other types of spatial orientation, are complex, intriguing and interrelated.

## Introduction

The strains of *Drosophila melanogaster* used in this study have been selected for positive and negative geotaxis since 1958 [1]. Hirsch and his students used recombination and chromosome substitution techniques to map multiple quantitative trait loci for geotaxis on each of the three large chromosomes (X, II, and III) of *D. melanogaster* [2,3]. They demonstrated that, in unselected (wild-type) *D. melanogaster*, genes on chromosomes X and II contributed mostly to positive geotaxis, and genes on chromosome III contributed to negative geotaxis [4]. They also reported a gene correlate of negative geotaxis near the Alcohol dehydrogenase gene (*Adh*, 2–50.1), indicating that *Adh* or a gene very near this locus enhances negative geotaxis in the *Hi5* geotaxis strain [5]. A strain not selected for geotaxis, but carrying the mutant spineless-aristapedia allele (*ss^a^*) which transforms the arista into tarsi, exhibits extremely positive geotaxis [6].

Using cDNA microarray and qPCR analyses, Toma and co-workers identified genes with differential mRNA expression in head extracts of Hirsch’s *Lo* and *Hi5* geotaxis strains [7]. Mutant alleles of the four genes exhibiting the most consistent mRNA differences between the geotaxis strains were transferred into wild-type (Canton-S) *D. melanogaster* to assay the effect of these alleles on geotaxis. Flies with mutations in three of these four genes, cryptochrome (c*ry^b^*, a strong hypomorph), Pigment-dispersing-factor (*Pdf^01^*, null-mutant) and Pendulin (*Pen^k14401^*, hypomorph) exhibited significantly altered geotaxis scores when compared to wild-type controls (Canton-S).

Both *cry* and *Pdf* are well documented as genes that control biological clocks in a variety of organisms. *cry* is involved in phase resetting (entrainment) of the circadian clock by blue light (<420 nm) phototransduction [8], but has no role in the core oscillator loop, responsible for circadian timing [9]. The mutant allele, *cry^b^*, disrupts normal phase response to shifted light cycles. *Pdf* plays a role in maintaining free-running circadian periods (*tau*) in constant darkness. *Drosophila* with P*df^01^* alleles tend to be arrhythmic or have short periods in constant darkness, and to have advanced activity peaks in light-dark, compared to the tau of flies with wild-type *Pdf* alleles [10].

Mertens and co-workers generated pigment dispersing factor receptor (P*dfr*) mutants in *D. melanogaster* using p-element insertions [11]. These P*dfr* mutants alter peptides that bind the *Pdf* gene product (PDF) to G protein-coupled receptors, which play an essential role in the cell-autonomous oscillator controlling circadian rhythms. Mutant *Pdfr*, inhibits the binding of PDF to its receptor. A mutation in either the *Pdf* or P*dfr* gene affects the function of the clock. The research of Mertens [11] parallels and supports Toma’s [7] conclusion that disrupting *Pdf* function increases negative geotaxis. See references [12,13] for more genes that affect geotaxis.

The hypotheses that the length of the adult, circadian, motor-activity cycles (tau) differ in *D. melanogaster* selected for positive and negative geotaxis response is tested and described below. The data presented and the literature reviewed in this paper demonstrate that strains of *D. melanogaster* selected for divergent geotaxis differ in characteristics of their biological clocks.

## Methods & Results

Results of statistical tests of difference between the sexes for circadian periods (tau), and for geotaxis, were not significant (p>0.05), so data of the sexes were combined for analysis. All statistical tests are two-tailed. All flies were maintained and tested at 24 +/– 1 ^o^C. All flies were kept in 12hr light:12hr dark (12L:12D) cycles prior to testing.

**Figure 1:**
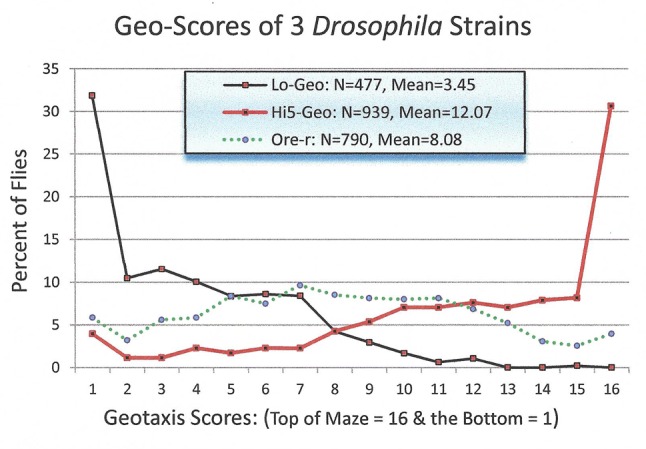
The distribution of *D. melanogaster Hi5* and *Lo* geotaxis strains, and the *Oregon-R (Ore-R)*, unselected, wild-type strain, in a 15-choice geo-maze. Flies finishing in exit tube “1” made 15 downward (geo-positive choices) and flies in tube “16” made 15 upward (geo-negative) choices. *Lo* and *Hi5* distributions were significantly different (X^2^_1_ = 507.2, p<0.001). The *Ore-R* data are included to illustrate the radical effect selection has had on the two selected, geotaxis strains. We can assume *Ore-R’s* response in the maze is similar to the strain Hirsch began working with in 1958.

A brief data set is provided (Figure 1) to document the *Lo* and *Hi5* strain’s geotaxis responses and to orientate readers not already familiar with their behavioural differences. Also included are geotaxis scores for the wild-type Oregon-R (*Ore-R*) strain of *Drosophila melanogaster*, to demonstrate the difference intense geotaxis selection has made in the *Lo* and *Hi5* strains. The original *Ore-R* flies were collected in 1925 by Lancefield in Roseburg, Oregon, USA and have been maintained in the laboratory without selection. *Ore-R* flies have been used in selection experiments [14], but the stock line has not, and remains wild-type.

### Geotaxis Methods

Flies were assayed for geotaxis in a geo-maze immediately after “lights on” in the 12 hour light:12 hour dark (12L:12D) cycle and maintained in constant light (L:L) until scored 24 hours later. The geo-maze is an enclosed 15 choice T-maze with arms of the T oriented vertically. Each choice proceeds through a stationary, cone-shaped gate that inhibits back-tracking [15]. Chi-square test for independence was used to test for difference between geotaxis scores of *Lo* and *Hi5* flies.

### Geotaxis Results

Figure 1 illustrates the geotaxis score distributions of the *Lo, Hi5 and Ore-R* strains. The differences between the three strains in the number of flies in tubes (1 to 8) compared to the number of flies in tubes (9 to 16) were all significantly different from each other. The mean geotaxis scores were, *Lo* (3.45), *Ore-R* (8.08) and *Hi5* (12.07) in the range of 1 to 16 maze end-tubes. Between the three strains there are only two independent comparisons. I tested the data sets combinations (*Lo* vs/ *Ore-R*) and (*Ore-R* /vs *Hi5*). Chi-square tests revealed highly significant differences in these comparisons, *X^2^*_(_*_df_*_=_*_1_*_)_* Lo/Ore-R* = 211.93; p<<0.001) and (*X^2^*_(_*_df_*_=_*_1_*_)_* Hi5/Ore-R* = 236.49: p<<0.001. From these results, we can infer that the third comparison (*Lo/Hi5*), showing the greatest difference, exhibited a significant *X^2^* result.

For comparison, the geotaxis scores Hirsch obtained from his wild-type, parent population (3 April, 1958), before selection of the *Lo* and *Hi5* geo-strains, were (males, *X^2^*= 7.27, N = 383, and female, *X^2^*= 7.23, N = 211) [Jerry Hirsch, personal communication, 2003].

### Methods for Determining Free-Running Periods of Adult Flies *(Tau)*

Flies were collected as late-stage pupae from stock vials following lights-on of a 12L:12D cycle. A Drosophila Activity Monitoring System (DAMS), obtained from Trikenetics Inc. (www.trikenetics.com), was used to record motor activity. Individual activity chambers were 5mm x 65mm glass tubes clipped between infrared emitter-detector pairs. A fly passing the detector at the mid-point of the tube broke the infrared beam and the time of activity was recorded in a computer file.

Pupae were placed in the glass test chamber, rather than adults, and emerged as adults 1-3 days later; consequently all eclosing flies were reliably virgin and of known age. Larva of nonvirginal females, moving about in the test chamber would invalidate the female’s recorded data. Pupae were harvested by flooding culture vials with water at 25 ^o^C and gently brushing them off the vial sides with a small, soft, artist’s paint brush. Pupae were removed from the water on plastic window screen and transferred to absorbent paper towelling where they were gently brushed to separate and dry them.

Approximately two cm of the activity-chamber tube was filled with agar-sucrose media and sealed to reduce evaporation by dipping the media-filled end into hot paraffin. After placing a single pupa into the tube, the open end was closed with a glued-on, paper disk, perforated with a needle to facilitate air diffusion. This end is usually plugged with a cotton wad, but in preliminary trials, I found that many newly eclosed flies became stuck in the cotton fibres by viscous body fluid that dried soon after eclosion; The DAMS monitors, with pupae, was placed in constant darkness (D:D) at the first scheduled lights-out of the 12L:12D cycle following their collection.

Activity counts were collected in six minute data bins (240 bins or data points/day) and stored in computer files for analysis. Records of the two days following the first activity record, which signalled eclosion of each fly, were discarded and the following ten days (2400 bins, data points) were used to determine each fly’s tau. Individual tau lengths and confidence levels were determined by Chi-square Periodogram Analysis [16,17]. The tau values ranged from 22 to 26 hours long.

Statistical tau determinations with p >0.01 were considered marginally unreliable and excluded from the data sets used to test for geotaxis strain difference. Chi Square Periodogram confidence levels were not used to test the hypothesis of difference between tau scores of the *Lo* and *Hi5* strains. Chi Square Periodogram confidence levels were used only to validate individual *tau* reliability for inclusion in data sets. One-way ANOVA for unequal sample size was used to test the hypothesis of difference between *Lo* and *Hi5* tau distributions.

### Results: Free-running Circadian Period of Adult Flies *(tau)*

**Figure 2:**
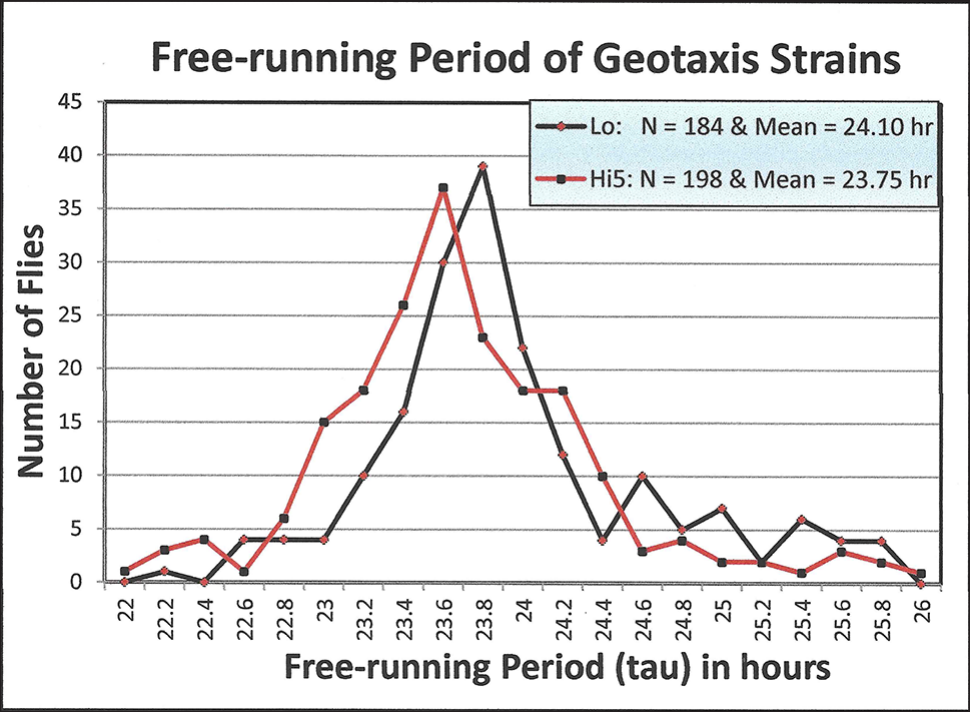
A comparison of the free-running activity period (tau) distribution of Hirsch’s *Lo* and *Hi5* strains of *Drosophila melanogaster*. The geo-positive strain (*Lo*) mean = 24.01 hours and the geo-negative strain (*Hi*)*5* mean = 23.75 hours, (ANOVA: F_1,380_ = 12.12, p<0.001). Activity records were obtained in constant darkness.

Figure 2 illustrates the difference in *tau* distributions of the *Lo a*nd *Hi5* strains in constant dark conditions. The *Lo* strain exhibited significantly longer tau (X = 24.1 hrs., N = 198) than the *Hi5* strain (X = 23.7 hrs, N = 184), (ANOVA: F_1,380_ = 12.12, P<0.001). Standard deviations of *Lo* and *Hi5* tau were not significantly different (*Lo* SD = 0.678 and *Hi5* SD = 0.706; F_s_ = 1.042, p>0.05).

## Discussion

Figure 1 contrasts the wild-type (*Ore-R*) geotaxis response to that of flies selected for positive and negative geotropism. The wild-type *Ore-R* flies have little up-down preference in the maze. The difference in free-running, circadian, activity-periods of *Lo* and *Hi5* geotaxis strains (Figure 2), was 24.1 and 23.7 hrs, respectively. Geotaxis and circadian behaviours are tied together in an interesting pleiotropic relationship as documented in this paper and others [7,11]. It is remarkable, then, that several investigators have failed to find circadian variation in geotaxis maze scores of wild-type *D. melanogaster* or in strains selected for geotaxis [7,12]. The contrast of the *Lo* and *Hi5* geo-flies with wild-type flies gives some understanding of the havoc artificial selection can play with gene pools adapted to natural conditions.

Hirsch periodically released subsets of the *Hi5* and *Lo D. melanogaster* lines from selection and observed regression toward wild-type geotaxis in following generations. But, after 21 years and 450 generations of selection, the lines became stable and did not regress when selection was relaxed [15]. This implies that the pressure of artificial selection has rearranged alleles into new adaptive gene complexes which provided new and stable behavioural expressions of geotaxis, adapted to the selection pressure of a geo-maze. This phenomena has been called “homeostatic fitness” [18,19].

Genetic homeostasis implies that not only have alleles of the genes affecting geotaxis been differentially selected in Hirsch’s *Lo* and *Hi5* lines; but compatible alleles of genes controlling adaptive and related behavioral, anatomical, or physiological traits, would be differentially selected as well. It is mind-boggling to imagine which alleles of which genes might enhance fitness when environmental conditions change; and what selective forces might be involved; but selection sorts it out over many generations. In the case of Hirsch’s flies, it took approximately 450 generations of rigorous artificial selection.

Wild-type *D. melanogaster* also orient in magnetic fields, but mutant (*cry^b^*), flies do not. The circadian clock of wild-type *D. melanogaster* is slowed (longer *tau*) in constant magnetic fields, in a dose dependent manner, but only in blue light and with a functional *cry* gene. Mutants with a hypomorphic cryptochrome allele (*cry^b^*) do not show this response to magnetic fields, whereas the clocks of mutants that over-express CRY, exhibit a greater than wild-type geotaxis response [10]. Interestingly, *cry*-dependent magnetosensitivity does not require a functioning circadian clock, but it does require a functional *cry* gene [20].

*cry’s* functional requirement for blue light (<420nm) in phase shifting circadian clocks and in altering spatial orientation and taxis in several species relative to gravity, magnetic fields, solar, lunar, and celestial radiation [21,22,23] makes it the most interesting of the genes currently associated with both biological clocks and geotaxis.

Ritz et al. [21,22] and Wiltschko et al., [23] present data showing that chickens, European robins, Australian silver eyes, newts and fruit flies use blue light-induced magnetoreception for orientation. These authors also present data to support a photo-induced radical-pair mechanism as the basis of the oriented response. A radical-pair is created by photo-induced electron transfer. The sensitivity of radical-pair reactions depends on the influence of static magnetic fields on spin states of the radical electron pair. For example, exposing chickens to an oscillating magnetic field of 1.566 MHz led to disorientation, suggesting disruption of the radical-pair mechanism. For a description of the radical-pair model see the Introduction of Yoshii et. al. [9].

The literature and the data presented here suggest that genes, physiology and behavioural aspects of geotaxis, circadian clocks, magnetosensitivity and spatial orientation are complex, intriguing and interrelated. Thiessen refers to behavior as a pleiotropic reflection of physiological processes. ‘Gene influence on behavior is always indirect’ [24, p.87]. Data contrasting the roles of genes controlling biological clocks, as well as geotaxis and other orientation phenomena provide a glimpse into the pleiotropic games genes play in shaping behaviors.

## Competing Interests

The author declares that they have no competing interests.
